# Post-traumatic stress disorder and its predictors in emergency medical service personnel: a cross-sectional study from Karachi, Pakistan

**DOI:** 10.1186/s12873-017-0140-7

**Published:** 2017-08-29

**Authors:** Salima Mansoor Kerai, Uzma Rahim Khan, Muhammad Islam, Nargis Asad, Junaid Razzak, Omrana Pasha

**Affiliations:** 1 Community Health Sciences Department, Aga Khan University, Karachi, Pakistan; 2 Emergency Medicine Department, Aga Khan University, Karachi, Pakistan; 3 Community Health Sciences Department, Aga Khan University, Karachi, Pakistan; 4 Psychiatry Department, Aga Khan University, Karachi, Pakistan; 5 Emergency Medicine Department, Johns Hopkins University, Baltimore, MD USA

**Keywords:** Emergency medical service, Low- and middle-income country, Karachi, Pakistan, Post-traumatic stress disorder

## Abstract

**Background:**

Emergency medical service (EMS) personnel who work to provide emergency medical care at the scene and during transportation are exposed to various kinds of stressors and are particularly susceptible to developing stress-reactions. This study assesses symptoms of post-traumatic stress disorder and its predictors among the personnel of a selected EMS in Karachi, Pakistan.

**Methods:**

Data were gathered from 518 personnel working in an EMS setting from February to May 2014. Participants were screened for post-traumatic stress symptoms using the Impact of Event Scale-Revised (IES-R). Demographic and work-related characteristics, coping styles and the social support systems of the participants were assessed. Linear regression was used on the IES-R to identify predictors of post-traumatic stress symptoms.

**Results:**

The mean score of the IES-R was 23.9 ± 12.1. EMS personnel with a dysfunctional coping style (β = 0.67 CI 0.39 – 0.95), anxiety, and depression (β = 0.64 CI 0.52 – 0.75) were more likely to have increased severity of post-traumatic stress symptoms. Age was found to have an inverse relationship with stress symptoms (β = −0.17 CI 0.33 – -0.023), indicating the susceptibility of younger EMS personnel to stress.

**Conclusion:**

The EMS personnel in this setting were found to have a moderate level of post-traumatic stress symptoms. The significant predictors of post-traumatic stress symptoms in this EMS population were age, coping style, and levels of anxiety and depression. These predicting factors can be a potential avenue for interventions to improve the mental health of these frontline workers.

## Background

Emergency medical service (EMS) personnel are exposed to a variety of work related stressors. These stressors range from critical incidents associated with the provision of patient care to chronic work-related problems (e.g., conflict with the supervisor, lack of support from colleagues, inadequate salary) [1]. Recurrent exposure to stressors may predispose EMS personnel to develop stress reactions such as burnout and post-traumatic stress disorder (PTSD) [1]. Therefore, it is critical to assess the mental health of EMS providers, who by the nature of their frontline work are at high risk for developing stress reactions.

A meta-regression analysis reveals that ambulance workers have the highest estimated prevalence (14.6%) among all occupational groups of rescue workers (like police, EMS, or firefighters) [2]. Data from high-income countries (HICs) indicate a wide range of PTSD prevalence (from 4 to 22%) among ambulance workers [3–5]. Unfortunately, there is a paucity of data related to mental health problems among EMS personnel from low- and middle-income countries (LMICs). The objective of this study is to assess post-traumatic stress symptoms and their predictors among EMS personnel in Karachi, Pakistan.

## Methods

### Study design and setting

This cross-sectional study was carried out in a pre-hospital setting from February to May 2014 in Karachi, Pakistan.

Karachi is one of the most densely populated metropolitan cities in the world, housing people from diverse ethnolinguistic backgrounds. Unlike cities in developed countries, there is no centralized EMS system in Karachi with a universal access number (UAN). Although the city has networks of ambulance services established by various private and not-for-profit organizations. Most of these ambulance services mainly provide transport to the hospital in a vehicle that has a siren, stretcher, and arrangement for oxygen supply, without any system of communication, neither with hospitals nor with medical personnel [6]. Apart from drivers who do not have any training, no EMS personnel is present onboard [6]. The study was carried out at a selected ambulance service, run by the AMAN foundation in Karachi. AMAN ambulance has EMS personnel (doctors, nurses, technicians) and drivers trained in lifesaving skills, and their ambulances are fitted with equipment and supplies to provide acute emergency medical care during transportation [7]. It is a tiered ambulance service with a third of ambulances classified as advanced life support (ALS) and the remaining two-thirds as basic life support. Only doctors and nurses with ALS training are deployed on the ALS ambulances. The organization has seven field stations and more than 80 ambulances on the road that work around the clock with extensive coverage in Karachi.

### Eligibility criteria

EMS personnel and drivers with at least 3 months of work experience on all three shifts (morning, evening, and night) were included in this study. This criterion was kept in order to have uniformity of field-level exposure to stressors.

### Measurement of PTSD

For assessment of post-traumatic stress symptoms, the Impact of Event Scale-Revised (IES-R) was used. It is a brief self-reporting tool, which has established utility for measuring post-traumatic stress symptoms in a variety of settings [8]. This 22-item scale consists of three subscales representing three symptom clusters: intrusion, avoidance, and hyper-arousal [8]. Participants reported their current level of symptomatic stress on a five point Likert scale (0–4) in response to any traumatic event/s experienced. No specific cut-off was used for analysis and interpretation of IES-R scores. The sum of means for each subscale has been recommended for reporting, as it allows the user to identify the degree of symptomatology [8], higher scores indicate greater symptomatic stress and a need for further evaluation [9]. The IES-R is the only validated tool in Urdu in Pakistan (with a reported good reliability (*Cronbach’s alpha* of 0.92) and satisfactory content and convergent validity for that sample [10]). The scale has been used by Ehring et al. in their studies for assessing PTSD in Pakistan (*Cronbach’s alphas* for their two studies were 0.91 and 0.94) [11, 12]. Prior to administration of the IES-R, the participants were asked to recall a traumatic event that they witnessed during the past week that was hurtful or terrifying for them. For the purpose of analysis, the responses were later coded as work related, not work related or no specific event recalled.

### Covariates

Data on covariates were collected, including socio-demographic (age, gender, income, marital status, family status, and education level) and work-related characteristics (type of job, length of service and average working hours per week). Social support, coping mechanisms, and problematic substance use were also assessed. For assessing social support, the Multi-Dimensional Scale of Perceived Social Support (MSPSS) was used. The MSPSS is a 12-item scale, with responses on a Likert scale of 1–7 to measure perceived social support from family, friends, and significant others (*Cronbach’s alpha* for each subscale was greater than 0.85 in our sample) [13]. For assessment of coping, the Brief COPE (coping inventory) scale was used; it is a 28-item tool to assess the frequency of different coping styles [14]. The factor structure of the Brief COPE was adopted from the second-order factor model from the study of people with Alzheimer’s disease and their caregivers (LASER-AD study) in which three subscales were reported: problem-focused coping (active coping, instrumental support, planning), emotion-focused coping (acceptance, emotional support, humor, positive reframing, religion), and dysfunctional coping (behavioral disengagement, denial, self-distraction, self-blame and substance abuse) [15]. We performed a confirmatory factor analysis to validate their factor structure in our data; we found consistency with a three-factor structure with model adequacy indices indicating a model with a fairly good fit (root mean square error of approximation (RMSEA), 0.063; comparative fit index (CFI), 0.818; and coefficient of determination (CD) 0.829). Total scores from the three subscales were calculated for the purpose of scoring (*Cronbach’s alpha* for each subscale ranges from 0.4 to 0.5 in our sample). Furthermore, a CAGE-AID (Cut down, Annoyed, Guilty, and Eye-opener-adapted to include drugs) questionnaire was used to screen for problematic substance use [16]. Problematic substance use was operationally defined as dependence on illicit or non-prescription drug use. Commonly used substances in our context were smoking and smokeless tobacco (such as mawa (tobacco with lime), paan (betel quid), and betel nuts). The family history of a mental health disorder, (such as depression, anxiety disorder, and sleep disorder) was assessed, along with a personal history of medication use to relieve psychological stress. These questions were asked with an underlying conjecture that people who have a family history of psychiatric morbidity or use medication to relieve stress are more susceptible to developing symptoms related to stress. Psychological stress was defined as any events that tax a person’s ability to cope adaptively, affecting their activities of daily routine [17]. Participants were also screened for anxiety and depression as co-existing conditions using the Aga Khan University Anxiety and Depression Scale (AKUADS). It is a 25-item tool that has been validated in Pakistan for screening anxiety and depression in communities [18].

### Data collection

Data were collected at field stations of the EMS. Lists of people working at field sites were obtained from station supervisors. In-person interviews were conducted with those who were eligible and gave consent to participate. A log of all personnel was maintained whether they consented to participate or not. Most of the data were collected during daytime hours, particularly during overlapping hours in the morning and evening shifts. The questionnaire was completed in approximately 20 min.

### Sample size

A calculated minimum of 384 participants was required in order to estimate the mean score of PTSD with a standard deviation of 19.75 [11], providing 2% precision, and a 5% level of significance. The total population working in AMAN ambulance at the time of the study was 536, which was greater than the calculated sample size. All EMS personnel and drivers were invited to participate in the study.

### Statistical analysis

In order to examine distributions, exploratory data analysis methods were used. Means and standard deviations were used for reporting of normally distributed continuous data. Categorical variables, such as gender and marital status, were reported in frequency and percentages. Data obtained from the IES-R tool were used in a continuous form. Linear regression was used to identify predictors of post-traumatic stress symptoms on the IES-R. Crude and adjusted beta coefficients with corresponding 95% confidence intervals were reported. Interaction and multi-collinearity were assessed. Model adequacy checks were performed. For statistical analysis, STATA version 12 was used (Copyright 1985–2011 Stata Corp LP).

## Results

**Fig. 1:**
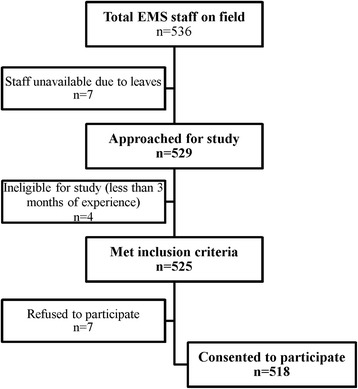
Study flow diagram

Out of 536 EMS personnel, 525 met the inclusion criteria, of whom 518 consented to participate (for details, see Fig. 1).

All field staff were male. Table 1 illustrates the socio-demographic profile of the surveyed EMS personnel in Karachi. A majority of the personnel were working as drivers (218, 42.1%) and nurses (205, 39.6%). The average age of the EMS personnel was 32 ± 6 years. The personnel were mostly married (376, 72.6%) and living in a joint-family system (436, 84.2%). The average years of work experience were 4 ± 1 years, with 47 ± 6.5 working hours per week. Most participants (413, 79.7%) had an average household monthly income between PKR 10,000 and 50,000. About a third of the participants (178, 34.3%) reported problematic substance use. More than half of the participants (278, 53.6%) reported that they had experienced a work-related traumatic event. Most of the EMS personnel predominantly used emotion-focused and problem-focused coping styles as compared to a dysfunctional coping style. EMS personnel were found to have higher perceived support from family (22.6 ± 4.8) in comparison to support from friends and significant others. One in six personnel (86, 16.6%) reported a family history of a mental health disorder, while only a few (27, 5.2%) reported medication use to relieve their psychological stress. The average score for anxiety and depression on the AKUADS was 15.6 ± 8.2.

**Table 1:** Socio-demographic profile of EMS personnel, Karachi, Pakistan

Variable	Results (*n* = 518)
Job types	
*Doctors*	38 (7.3%)
*Nurses*	205 (39.6%)
*Drivers*	218 (42.1%)
*Technicians*	57 (11.0%)
Age groups in years	
≤ 25	58 (11.2%)
26–35	352 (68.0%)
36–45	97 (18.7%)
≥ 46	11 (2.1%)
Marital status	
Single/Separated	142 (27.4%)
Married	376 (72.6%)
Living status	
Alone	82 (15.8%)
Family	436 (84.2%)
Type of family (*n* = 436)	
*Extended family*	278 (63.8%)
*Nuclear family*	158 (36.2%)
Years of experience (Mean ± SD)	4 ± 1.0
Average working hours in a week (Mean ± SD)	47.2 ± 6.5
Years of formal schooling (Mean ± SD)	13.8 ± 2.9
Average household monthly income in PKR	
10,000–25,000	201 (38.8%)
25,000–50,000	212 (40.9%)
50,000–100,000	89 (17.2%)
100,000–300,000	16 (3.1%)
Substance use problem	178 (34.3%)
Type of traumatic event	
Work related	278 (53.6%)
Not work related	79 (15.2%)
None specified	161 (31.0%)
Coping style (Mean ± SD)	
Dysfunctional	17.5 ± 3.3
Emotion focused	24.5 ± 3.9
Problem focused	25.3 ± 3.2
Perceived social support (Mean ± SD)	
Family	22.6 ± 4.8
Friends	19.9 ± 5.1
Significant others	19.7 ± 7.3
Family history of mental health disorder	86 (16.6%)
Personal use of medicine to relieve stress	27 (5.2%)
Post-traumatic stress disorder (Mean ± SD)	
IES-R score	23.9 ± 12.1
Anxiety and depression (Mean ± SD)	
AKUADS score	15.6 ± 8.2

The crude overall mean score of post-traumatic stress symptoms on the IES-R was 23.9 ± 12.1. The mean of each subscale was: 1.2 ± 0.6 for intrusion, 1.0 ± 0.6 for avoidance, and 0.8 ± 0.6 for hyper-arousal. The sum of means of each subscale was 3 ± 1.6 (data not shown in table).

Table 2 models the predictors of post-traumatic stress symptoms using the least square method. An inverse relationship was seen between age and symptom severity (β = −0.17, 95% CI -0.33–0.023, *P* = 0.03). This implies that young people are more vulnerable to stress. Moreover, people who use dysfunctional coping were more likely to have an increased symptom severity (β = 0.67, 95% CI 0.39–0.95, *P* < 0.05). Likewise, EMS personnel who have higher scores for anxiety and depression have higher levels of post-traumatic stress symptoms (β = 0.64, 95% CI 0.52–0.75, *P* < 0.05). No statistically significant differences were detected among other variables. Errors were heteroskedastic for this estimated model, i.e., they showed no constancy of error variance. Moreover, no interaction or collinear variable was found.

**Table 2:** Predictors of post-traumatic stress symptoms among EMS personnel, Karachi, Pakistan

	Adjusted β coefficient	95% confidence interval	*P*
Age (in years)	−0.17	−0.33 – −0.023	0.03
Dysfunctional coping	0.67	0.39 – 0.95	< 0.05
Symptoms of anxiety and depression	0.64	0.52 – 0.75	< 0.05

R^2^ for the model is 28%

RMSEA is 10.34

## Discussion

The crude mean score of post-traumatic symptoms on the IES-R was 23.9 ± 12.1, with a sum of means of 3 ± 1.6. This indicated a moderate level of symptomatic stress among EMS personnel in Karachi, Pakistan. Symptom severity indicated by mean score was relatively higher in this study population when compared to a similar population in Mansoura (Egypt) and rescue workers in Lahore (Pakistan), and Western Cape province (South Africa) [11, 19, 20]. This variation in estimates is attributable to several reasons but primarily to differences in population and context. This study was conducted only on EMS personnel, while previous research focused on mixed populations, including EMS personnel, firefighters, and police. Background literature has mostly looked at EMS along with other rescue workers and has shown that the stress and burnout of EMS personnel is the highest compared to other rescue workers [2]. EMS personnel particularly work around people with trauma or severe injuries or those who are at high risk of dying. Chronicity of exposure to traumatic events and critical incidents in this population can be considered as more pervasive and persistent as compared to other occupational groups. Of note, there is a lack of institutional scaffolding related to rescue service and disaster management in the country (except for Punjab province) [21, 22]. Rescue services like firefighters and ambulance services are mostly operated by non-state organizations, with varying job responsibilities, work milieu and coverage, and no inter-agency coordination even in times of emergencies or disaster. Due to heterogeneity in their job demands, it can be assumed that the exposure of dealing with traumatic events would be different for each service, leading to varied stress responses. Screening for mental health morbidity based on differential exposure between the occupational groups was out of the scope of our work, but it is a potential area to be explored for future studies.

Variation in the estimates of levels of stress reactions can also be attributed to differences in the instruments used in Karachi and Mansoura. In addition, the socio-political context in Karachi differs considerably from the above-mentioned research settings. This study was conducted around times of increased violence and strife within Karachi [23]. Data suggest that among all South Asian countries, Pakistan has the highest number of fatalities due to terrorism, with Karachi’s having one of the highest recorded figures [23, 24]. Results from a study conducted in four emergency departments (EDs) of Karachi showed that 16.5% of nurses and physicians reported physical attacks, and 72.5% reported verbal abuse [25]. While it is difficult to assess the contribution of individual direct exposure and the baseline indirect exposure, we believe that direct exposure to violent and traumatic incidences is likely to have had a greater impact on the individual’s level of stress and mental health.

The study also identified some important predictors of post-traumatic stress symptoms among the study participants. Younger EMS personnel were more likely to have increased severity of symptoms. This seems plausible because younger adults may be less able to manage stress. On the other hand, the findings showed that participants with a dysfunctional coping style had an increased level of post-traumatic stress symptoms. Dysfunctional coping is generally viewed as an avoidant coping mechanism due to the adoption of maladaptive coping techniques [26]. Studies have shown that dysfunctional coping can predict complicated grief and PTSD severity, which could potentially worsen mental well-being [26]. In this community, these findings require special attention due to lack of resources and difficulty experienced by Pakistani men in expressing pain and stress symptoms. This may predispose the personnel to resort to dysfunctional coping mechanisms in stressful situations. Our study suggests the need for educating these frontline workers about coping mechanisms and stress management techniques to prevent adverse mental health conditions. The study also showed that those personnel who have higher levels of post-traumatic stress symptoms have higher scores for anxiety and depression. One possible explanation could be a pre-existing negative appraisal of life events, which tends to get intensified with exposure to stressors.

### Limitations and strengths

The evidence for the validity of screening tools, especially from the LMIC context, is scarce, hence it was advisable to continue the use of the most widely used and validated tool from local setting [27]. Due to resource limitation, we were unable to use the gold standard test which is a clinician-based diagnostic interview, thus IES-R was used. It is widely used for screening of post-traumatic stress symptoms across various population groups and has been translated into many languages [8]. There are several cutoff values under discussion for establishing a probable diagnosis of PTSD ranging from 22 in people with substance use [28] to 33 in Vietnam veterans [29]. However, these cutoff values were based on specific population groups and context; they were not applicable in this setting. Besides, as it is a descriptive tool intended to provide an indication of the general level of symptomatic stress related to an event, not to establish a diagnosis [9], cut-off points seemed inappropriate.

Another possible limitation was related to the CAGE-AID tool used for screening of problematic substance use. This is a blunt tool, where only two possible responses (yes and no) are available. Hence, higher scores indicate only the presence of a substance use problem, not the intensity (frequency/quantity/consumption) or type of drug used. Neither does this tool discriminate between active or inactive substance users. In addition, there is a stigma attached to the term “substance abuse” in the local setting, making the authors cautious in the use of the term. It was observed that smoking was the most commonly used substance as compared to alcohol or other drugs (commonly defined as marijuana, heroin or cocaine in the local setting).

In addition, there might be a problem of social desirability bias whereby people respond in a socially acceptable manner. The interviewers, however, were unknown to the respondents; they were independent people who had no affiliation with the participating institution or with its mission or employees. Interviews were conducted in a private closed room. No one from the institution had access to the logs of participation other than the study team.

Moreover, there might be underreporting of psychological symptoms, which could have led to an underestimation by study estimates. This is generally a problem with studies focused on mental health, since mental health is often not considered an integral part of overall well-being. Additionally, under-reporting may have occurred because mental health illnesses are a source of social stigma. While the recall period in the study was not greater than 2 weeks, there could have been recall limitations in which participants might have had difficulty in recalling their experiences. Also, there may have been a problem of length bias sampling. Those who had post-traumatic stress symptoms for a long time would have been captured by the screening tool, whereas those who spontaneously recovered or left the job due to increased levels of symptomatic stress might have been left out of the sampling frame.

For the sake of this study, we included the only ambulance service in Karachi that provides emergency medical care at the scene and during transportation. There are several other ambulance services that provide efficient taxi services for ill/injured patients with little or no lifesaving equipment and are staffed by medically untrained drivers. All EMS personnel including drivers were male in our study. One of the main reason for male dominant workforce is the socio-cultural context of the Pakistani society that discourages women participation in outdoor work specially in high risk occupational group. Our work is generalizable to ambulance services that are involved in medical service provision.

The literature on mental health problems amongst EMS personnel is scarce. This is one of the very few studies to assess a mental health condition like PTSD in an EMS community from one of the LMIC like Pakistan.

## Conclusion

Within limitations, it can be concluded that the EMS population in this setting has a moderate level of post-traumatic stress symptoms. Statistically significant predictors of post-traumatic stress symptoms in this EMS population include age, coping style, and levels of anxiety and depression. EMS institutions should have measures in place to identify and screen high-risk people, together with providing targeted interventions to those in need. There is also a need to direct resources toward the provision of mental health services for promoting psychological and mental well-being in this high-risk group.

## Abbreviations

AKUADS: Aga Khan University Anxiety and Depression Scale
ALS: Advanced Life support
CAGE-AID: Cut down, Annoyed, Guilty, and Eye-opener-adapted to include drugs
CD: Coefficient of Determination
CFI: Comparative Fit Index
EDs: Emergency department
EMS: Emergency medical service
HICs: High-income countries
IES-R: Impact of Event Scale-Revised
LMICs: Low- and middle-income countries
MSPSS: Multi-Dimensional Scale of Perceived Social Support
PTSD: Post- Traumatic Stress Disorder
RMSEA: Root Mean Square Error of Approximation
UAN: Universal access number

## References

[1] Donnelly E (2012). Work-related stress and posttraumatic stress in emergency medical services. Prehosp Emerg Care.

[2] Berger W, Coutinho ESF, Figueira I, Marques-Portella C, Luz MP, Neylan TC (2012). Rescuers at risk: a systematic review and meta-regression analysis of the worldwide current prevalence and correlates of PTSD in rescue workers. Soc Psychiatry Psychiatr Epidemiol.

[3] Sterud T, Ekeberg O, Hem E (2006). Health status in the ambulance services: a systematic review. BMC Health.

[4] Misra M, Greenberg N, Hutchinson C, Brain A, Glozier N (2009). Psychological impact upon London ambulance service of the 2005 bombings. Occup Med.

[5] Mishra S, Goebert D, Char E, Dukes P, Ahmed I (2010). Trauma exposure and symptoms of post-traumatic stress disorder in emergency medical services personnel in Hawaii. Emerg Med J.

[6] Baqir M, Ejaz K (2011). Role of pre-hospital care and ambulance services in Karachi. J Pak Med Assoc.

[7] Aman Ambulance. The Aman Foundation. 2016. https://www.theamanfoundation.org/program/aman-ambulance/. Accessed 28 Aug 2017.

[8] Weiss DS. The Impact of Event Scale: Revised. In: John P. Wilson CCS-KT, editor. Cross-Cultural Assessment of Psychological Trauma and PTSD. Springer Science & Business Media; 2007.

[9] Motlagh H (2010). Impact of event scale-revised. J Physiother.

[10] Tareen MS, McDowell C, Naqvi K, Bashir A, Keenan P, Rehman Au, et al. Evaluation of an Urdu version of the Impact of Event Scale – Revised. International Psychiatry. 2012;9(1)​:20–2.

[11] Razik S, Ehring T, Emmelkamp PM. Psychological consequences of terrorist attacks: Prevalence and predictors of mental health problems in Pakistani emergency responders. Psychiatry Res. ​2013;207(1):80–5. 10.1016/j.psychres.2012.09.031 23068079

[12] Ehring T, Razik S, Emmelkamp PM (2011). Prevalence and predictors of posttraumatic stress disorder, anxiety, depression, and burnout in Pakistani earthquake recovery workers. Psychiatry Res.

[13] Nancy W, Dahlem GDZ, Robin R (1991). Walker. The multidimensional scale of perceived social support: a confirmation study. J Clin Psychol.

[14] Carver CS (1997). You want to measure coping but your Protocol's too long: consider the brief COPE. Int J Behav Med.

[15] Cooper C, Katona C, Livingston G (2008). Validity and reliability of the brief COPE in carers of people with dementia: the LASER-AD study. J Nerv Ment Dis.

[16] Brown RL, Rounds LA (1994). Conjoint screening questionnaires for alcohol and other drug abuse: criterion validity in a primary care practice. Wis Med J.

[17] Cohen S, Janicki-Deverts D, Miller GE (2007). Psychological stress and disease. JAMA.

[18] Karmaliani R, Bann CM, Pirani F, Akhtar S, Bender RH, Goldenberg RL (2007). Diagnostic validity of two instruments for assessing anxiety and depression among pregnant women in Hyderabad, Pakistan. Health Care Women Int.

[19] Ward C, Lombard C, Gwebushe N (2006). Critical incident exposure in south African emergency services personnel: prevalence and associated mental health issues. Emerg Med J.

[20] Khashaba EO, El-Sherif MA, Ibrahim AA, Neatmatallah MA (2014). Work-related psychosocial hazards among emergency medical responders (EMRs) in Mansoura City. Indian J Community Med.

[21] Sasser S, Varghese M, Kellermann A, Lormand JD (2005). Prehospital trauma care systems.

[22] Cheema AR, Mehmood A, Imran M (2016). Learning from the past: analysis of disaster management structures, policies and institutions in Pakistan. Disaster Prev Manag.

[23] South Asia Fatalities. In: South Asia Terrorism Portal. South Asia Intelligence review. 2014. http://www.satp.org/satporgtp/southasia/datasheets/Fatalities.html. Accessed 28 Aug 2017.

[24] Bhatti JA, Mehmood A, Shahid M, Bhatti SA, Akhtar U, Razzak JA (2011). Epidemiological patterns of suicide terrorism in the civilian Pakistani population. Int J Inj Control Saf Promot.

[25] Zafar W, Siddiqui E, Ejaz K, Shehzad MU, Khan UR, Jamali S (2013). Health care personnel and workplace violence in the emergency departments of a volatile metropolis: results from Karachi, Pakistan. The Journal of Emergency Medicine.

[26] Schnider KR, Elhai JD, Gray MJ (2007). Coping style use predicts posttraumatic stress and complicated grief symptom severity among college students reporting a traumatic loss. J Couns Psychol.

[27] Ali GC, Ryan G, De Silva MJ (2016). Validated screening tools for common mental disorders in low and middle income countries: a systematic review. PLoS One.

[28] Rash CJ, Coffey SF, Baschnagel JS, Drobes DJ, Saladin ME (2008). Psychometric properties of the IES-R in traumatized substance dependent individuals with and without PTSD. Addict Behav.

[29] Creamer M, Bell R, Failla S (2003). Psychometric properties of the impact of event scale-revised. Behav Res Ther.

